# Discovery and characterization of a high-affinity and high-specificity peptide ligand LXY30 for in vivo targeting of α3 integrin-expressing human tumors

**DOI:** 10.1186/s13550-016-0165-z

**Published:** 2016-02-27

**Authors:** Wenwu Xiao, Tianhong Li, Fernanda C. Bononi, Diana Lac, Ivy A. Kekessie, Yanlei Liu, Eduardo Sanchez, Anisha Mazloom, Ai-hong Ma, Jia Lin, Jimmy Tran, Kevin Yang, Kit S. Lam, Ruiwu Liu

**Affiliations:** Department of Biochemistry and Molecular Medicine, University of California Davis, Sacramento, CA 95817 USA; Division of Hematology/Oncology, Department of Internal Medicine, University of California Davis, Sacramento, CA 95817 USA; University of California Davis Comprehensive Cancer Center, Sacramento, CA 95817 USA; Veterans Affairs Northern California Health Care System, Mather, CA 95655 USA; Department of Pathology and Laboratory Medicine, University of California Davis, Sacramento, CA 95817 USA; The College of the University of Chicago, Chicago, IL 60602 USA

**Keywords:** Cancer-targeting peptide, α3β1 integrin, One-bead one-compound combinatorial peptide library, Glioblastoma, Optical imaging

## Abstract

**Background:**

α3β1 integrin is overexpressed in several types of human cancer and is associated with poor prognosis, metastasis, and resistance to cancer treatment. We previously identified a cyclic peptide ligand LXY1 that specifically binds to the α3β1 integrin on human glioblastoma U-87MG cells. Here, we optimized LXY1 through one-bead one-compound combinatorial library screening and site-specific modifications to improve its in vivo binding property.

**Methods:**

Three bead libraries were synthesized and whole-cell binding assays were performed. The binding capacity of individual peptide ligands against different tumor cells was determined by flow cytometry and confirmed by optical imaging. A complex joining biotinylated ligand with streptavidin-Cy5.5 was used for in vivo target imaging in both subcutaneous and orthotopic U-87MG xenograft mouse models.

**Results:**

LXY30, a cyclic peptide with the sequence cdG-Phe(3,5-diF)-G-Hyp-NcR, emerged as the most potent and selective ligand for the α3 subunit of α3β1 integrin with improved in vitro and in vivo tumor-targeting effects compared to LXY1 in U-87MG cells. LXY30 is considerably stable in plasma as demonstrated in an in vitro stability study in 90 % human plasma. LXY30 also binds to several other known α3β1 integrin-expressing glioblastoma, lung, and breast cancer cell lines with various affinities.

**Conclusions:**

Our data support further investigating the role of LXY30 as a human tumor-targeting peptide ligand for systemic and intracranial delivery of imaging agents and cancer therapeutics.

**Electronic supplementary material:**

The online version of this article (doi:10.1186/s13550-016-0165-z) contains supplementary material, which is available to authorized users.

## Background

Integrins are a family of heterodimeric transmembrane glycoproteins that are overexpressed in various cell types, including angiogenic endothelial cells and certain cancer cells. Integrins are involved in a wide range of cell-to-extracellular matrix (ECM) and cell-to-cell interactions, mediating cell adhesion, signal transduction, tumorigenesis, tumor growth, and metastasis [1, 2]. Thus, integrins are attractive targets for the treatment and prevention of several diseases, including cancer [3]. They are transmembrane αβ heterodimers and so far 18 α and 8 β subunits are known in humans, generating 24 heterodimers. To the best of our knowledge, among the 8 β subunits, β1 is the sole β subunit to form heterodimer with α3. The generated α3β1 integrin is a laminin receptor with diverse biological functions. In epithelial cells, it acts as a receptor for the basement membrane, whereas in neuronal and tumor cells, it mediates migration. α3β1 integrin plays an important role in normal development of the lung, kidney, cerebral cortex, epithelium, liver, and muscle [4]. Overexpression of α3β1 integrin has been reported in several cancer types, such as glioblastoma [5], ovarian cancer [6], breast cancer [7–9], lung cancer [10], and melanoma [4] and has been associated with poor prognosis, tumorigenesis, tumor invasion, metastasis, and resistance to cancer treatment [11–14]. Thus, α3β1 integrin has been investigated as a promising cancer-specific biomarker and pharmacological target.

Our group has previously identified and characterized several tumor-targeting peptide ligands against a variety of integrins, including α3β1 [15, 16], α4β1 [17], and αvβ3 [18], using the high-throughput one-bead one-compound (OBOC) combinatorial library approach [19, 20] and an “on-bead” whole-cell binding assay [15]. We found a peptide motif [c(d/D)GXGXXc] (where d-amino acids are denoted by lowercase and X represents a randomized position during combinatorial library synthesis) for α3β1 integrin-expressing ovarian adenocarcinoma, glioblastoma, and metastatic breast cancer [15] and a peptide motif [cNGXGXXc] for α3β1 integrin-expressing non-small cell lung cancer (NSCLC) [21]. One of these lead ligands is LXY1 [cdGLG-Hyp-Nc], where Hyp is hydroxyproline, a cyclic peptide ligand that binds to α3β1 integrin on human glioblastoma U-87MG cells [16]. We performed a series of structure-activity relationship (SAR) studies to optimize LXY1 and identified two peptide ligands LXY3 [cdG-Tyr(3-NO_2_)-G-Hyp-Nc] [22] and LXY4 [cdG-Phe(3,5-diF)-G-Hyp-Nc] [23] (previously named analog 29 in the original report), which, compared to LXY1, had comparable and improved in vitro binding affinity to U-87MG cells as well as human breast cancer MDA-MB-231 cells. Yet neither LXY3 nor LXY4 showed improved in vivo tumor-targeting property compared with LXY1, limiting their translational application in cancer diagnosis and treatment. The poor in vivo tumor targeting may be due to the rapid degradation of integrin ligands by metabolism and/or their suboptimal binding to α3β1 integrin on tumor cells in living animals. The objective of this study was to further optimize the peptide ligands for α3β1 integrin-expressing human cancer cells for use in in vivo imaging and therapeutic studies.

We hypothesized that further optimization of LXY1 by modifying individual amino acids could improve its in vivo binding affinity and stability to α3β1 integrin-expressing human cancer xenografts. In order to optimize the binding affinity of tumor-targeting peptide ligands against α3β1 integrin, we employed two complementary approaches in tandem, a focused OBOC combinatorial library screening method followed by traditional medicinal chemistry modification. We identified LXY30 [cdG-Phe(3,5-diF)-G-Hyp-NcR] as the most potent ligand against α3β1 integrin on glioblastoma U-87MG cells. Antibody blocking demonstrated that LXY30 bound to the α3 subunit of α3β1 integrin. We further demonstrated that LXY30 has better in vitro and in vivo tumor-targeting abilities than LXY1. We subsequently confirmed the binding of LXY30 to a panel of breast and NSCLC cells. All data support further investigation of LXY30 as a common tumor-targeting peptide ligand for systemic and intracranial delivery of diagnostic imaging agents and cancer therapeutics.

## Methods

### Materials

TentaGel S NH_2_ resin (90 μm, 0.26 mmol/g) was purchased from Rapp Polymere GmbH (Tϋbingen, Germany). Rink amide MBHA resin (0.5 mmol/g) was purchased from GL Biochem (Shanghai, China). 6-chloro-*N*-hydroxybenzotriazole (6-Cl HOBt), Fmoc-Lys(Biotin)-OH, Fmoc-Lys(Dde)-OH, and Fmoc-natural amino acids were purchased from AAPPTec (Louisville, KY). Fmoc-unnatural amino acids were purchased from Chem-Impex International, Inc. (Wood Dale, IL). CLEAR-OX™ resin was purchased from Peptide International Inc (Louisville, KY). 1,3-Diisopropylcarbodiimide (DIC), trifluoroacetic acid (TFA), fluorescein isothiocyanate (FITC), all solvents, and other chemical reagents were purchased from Aldrich (Milwaukee, WI) and were analytical grade. Matrix-assisted laser desorption/ionization time of flight mass spectrometry (MALDI-TOF MS) analysis was performed on a Bruker BIFLEX III mass spectrometer (Billerica, MA). Analytical HPLC was performed on a Waters 2996 HPLC system equipped with a 4.6 × 150 mm Waters Xterra MS C18 5.0 μm column and employed a 20-min gradient from 100 % H_2_O/0.1 % TFA to 100 % acetonitrile (ACN)/0.1 % TFA at a flow rate of 1.0 mL/min. Preparative HPLC was performed on a System Gold 126NMP solvent module (Beckman) with a C18 column (Vydac, 10 μm, 2.2 cm i.d. × 25 cm). A gradient elution of 0–60 % B over 45 min, then 60–100 % B over 5 min followed by 100 % B for 5 min was used at a flow rate of 5 mL/min (solvent A, H_2_O/0.1 % TFA; B, ACN/0.1 % TFA). Anti-α3 and anti-β1 integrin antibodies were purchased from Chemicon International, Inc. (Billerica, MA). Glioblastoma, breast cancer, and lung cancer cell lines were obtained from American Type Culture Collection (Manassas, VA). U-87MG transfected with luciferase was purchased from Xenogen Corporation (Alameda, CA).

### Synthesis of focused OBOC peptide libraries

The OBOC libraries were synthesized on TentaGel S NH_2_ resin (0.26 mmol/g) with Fmoc-chemistry employing a “split-mix” strategy using 6-Cl HOBt/DIC as coupling reagents [16–18]. A four-fold molar excess of Fmoc-protected amino acids to resin was used for coupling. Each coupling reaction took place at room temperature for 2–6 h, considered complete when the ninhydrin test was negative. The Fmoc group was deprotected with 20 % 4-methylpiperidine in DMF (first 5 min, then 15 min). After the last cycle of amino acid coupling and Fmoc-deprotection, the side chain protecting groups were removed with TFA cocktail containing 82.5 % TFA, 5 % phenol, 5 % thioanisole, 5 % H_2_O, and 2.5 % triisopropylsilane (TIS). The disulfide formation was achieved with 20 % DMSO in ammonia acetate buffer (pH 6.2) for 2 days. Ellman’s test was negative. The selection of amino acids for each position is presented in Additional file 1: Figures S1 and S2, except position X_2_ that only has two β-turn inducing amino acids (Hyp and P).

### “On-bead” whole-cell binding assay

U-87MG cells adherent to the bottom of a T75 flask were trypsinized with 0.05 % trypsin-EDTA and neutralized with culture medium. Floating cells were collected, centrifuged, and resuspended in 10 mL of culture medium in a 10-cm Petri dish. During each screening, 50 μL of OBOC library beads were washed sequentially with ethanol, water, and phosphate-buffered saline (PBS). The beads were then incubated with suspended U-87MG cells, and the entire dish was swirled at a speed of 40 rpm inside an incubator at 37 °C under 5 % CO_2_. The plate was examined for cell-bead binding under an inverted microscope every 15 min.

### Synthesis of peptide, peptide-biotin, and peptide-FITC conjugates

To construct the peptides on Rink amide MBHA resin (loading 0.5 mmol/g), we employed a standard solid-phase peptide synthesis approach using Fmoc-tBu chemistry and 6-Cl HOBt/DIC coupling [16, 17]. A four-fold molar excess of Fmoc-protected amino acids to resin was used for coupling. The reaction was monitored with the ninhydrin test. The Fmoc group was deprotected with 20 % 4-methylpiperidine in DMF (first 5 min, then 15 min). The peptides were cleaved from Rink resin using a TFA cocktail as described above, followed by precipitation with cold diethyl ether. Crude peptides were dissolved in 50 % 0.1 M ammonium acetate buffer in ACN and cyclized with CLEAR-OX resin at room temperature for 1–3 h [24]. Ellman’s test was negative to confirm reaction completion. The liquid was collected by filtration. The beads were washed with a small amount of 50 % ACN/water. The combined solution was lyophilized to obtain powder. The powder was re-dissolved in small amount of 50 % ACN/water and then purified by RP-HPLC on a preparative Vydac C18 column. The purity was determined to be >95 %. The identities of peptides were confirmed with high-resolution electrospray ionization mass spectrometry (HR ESI-MS), and the data are shown in Additional file 1: Table S1. The ^1^H NMR of LXY30 is described in Additional file 1 (Method S1).

Synthesis of peptide conjugates was similar to the method described above. Peptide-biotin and peptide-FITC were designed to have biotin or FITC attached to the side chain of Lys and two hydrophilic linkers between peptide and Lys (biotin) and Lys (FITC). The synthesis was performed on Rink amide MBHA resin. The synthetic scheme is shown in Fig. 2. Fmoc-Lys (Dde)-OH was first coupled to the resin, followed by coupling of two linkers. Then, peptides were constructed as described above on the N-terminus of the linker. The Dde-protecting group was removed with 2 % NH_2_NH_2_ in DMF twice (5, 10 min). The beads were washed with DMF, MeOH, and DMF, followed by the addition of biotin (3 eq. to resin), HBTU (3 eq.), and DIEA (6 eq.) in 1-methyl-2-pyrrolidone for peptide-biotin, or FITC (3 eq.) and DIEA (6 eq.) in DMF for peptide-FITC. The coupling reaction was conducted at room temperature overnight. The cleavage and cyclization were achieved as described above. The purity was determined to be >95 %. The identity of the compounds was confirmed by MALDI-TOF MS (Additional file 1: Table S3).

### Flow cytometry

Confluent tumor cells (80–90 %) were dissociated with 0.05 % trypsin-EDTA and neutralized with culture medium. To determine the expression of α3 and β1 integrins, 1 μg of anti-α3 or anti-β1 antibody was mixed with 1 million cells for 30 min, followed by anti-mouse IgG-PE incubation and flow cytometry (Coulter XL-MCL). To demonstrate the peptides’ binding affinity, the cells (3 × 10^5^) in each sample were incubated with biotinylated peptides in 50 μL of PBS containing 10 % FBS and 1 mM MnCl_2_ for 30 min on ice. Then each sample was washed three times with 1 mL PBS containing 1 % FBS. Samples thereafter were incubated with a 1:500 dilution of streptavidin-PE (1 mg/mL) for 30 min on ice followed by a single wash with 1 mL of PBS containing 1 % FBS. Finally, the samples in PBS were analyzed by flow cytometry. To determine the relative binding affinity among different peptides, 0.4 μM of each non-biotinylated peptide including LXY1 was separately mixed first with 0.5 μM biotinylated LXY1. The mixture was then incubated with cancer cells followed by streptavidin-PE. Samples were analyzed by flow cytometry, and mean fluorescence intensity (MFI) was decided for each individual sample. The relative competition effect compared to LXY1, named as relative binding index (RBI), was evaluated with the following formulation: (MFI of positive control−MFI of the sample)/(MFI of positive control−MFI of LXY1), where the positive control was 0.5 μM biotinylated LXY1 only without competition. For blocking experiments, the samples were incubated with non-biotinylated LXY30, anti-α3, or anti-β1 antibody for 30 min prior to incubation with 20 nM biotinylated LXY30. For binding affinity measurement by direct conjugation of peptide and fluorophore, 0.5 μM scrambled LXY30-FITC (S-LXY30-FITC) or LXY30-FITC was incubated with 3 × 10^5^ U-87MG cells for 30 min, then the cells were washed with PBS containing 1% FBS and examined by flow cytometry. The binding strength of biotinylated LXY1, LXY4, LXY7, and LXY30 against several human glioblastoma, breast cancer, and lung cancer cell lines was determined using 1 μM biotinylated peptide.

### In vitro fluorescence and confocal microscopy

For the cell line staining, the samples were incubated with 1 μM scrambled LXY30-FITC (S-LXY30-FITC) or LXY30-FITC for 30 min on ice. After the wash, the cells were resuspended in 100 μL of PBS and loaded into a cytospin centrifuge vial (Cytospin 3; Shandon). The cells were centrifuged onto slides at 300×*g* for 2 min, mounted using a DAPI-containing fluorescence mount solution (Invitrogen), and examined under a fluorescence microscope (IX81; Olympus) (image software: Metaphore). For confocal microscopy, U-87MG cells adhering on the bottom of the chamber slides were incubated with 1 μM biotinylated LXY30 streptavidin-Alexa488 conjugates or negative control G6 peptide for 2 h and then observed under an LSM710 confocal fluorescence microscope (Zeiss). For the microscopic evaluation of xenografts, 10-μm cryosections of orthotopic or intracranial and subcutaneous U-87MG tumor were fixed in acetone at −20 °C for 20 min. After washing with PBS, the sections were mounted and observed as described as above.

### Tumor xenografts

Animal studies were performed according to a protocol approved by IACUC of the University of California, Davis. Female athymic nude mice (nu/nu), obtained from Harlan (Indianapolis, IN) at 5–6 weeks of age, were injected subcutaneously in the right flank with 5 × 10^6^ U-87MG cells. For orthotopic or intracranial implantation, 2.5 × 10^5^ cells in 5 μL PBS were injected into the right striatum area of the mouse with the aid of a mouse stereotactic instrument (Stoelting). When the subcutaneous tumors reached 0.5 to 1.0 cm in diameter or 21–28 days after implantation (at that time, the mean size of orthotopic or intracranial xenograft tumors was 0.2 to 0.5 cm in diameter), the tumor-bearing mice were subjected to in vivo and ex vivo imaging studies.

### In vivo and ex vivo optical and bioluminescence imaging

Biotinylated peptide-streptavidin (SA)-Cy5.5 (1.8 nmol), prepared by mixing 7.2 nmol of biotinylated peptide with 1.8 nmol of streptavidin-Cy5.5 in PBS overnight at 4 °C, was injected via the tail vein in an anesthetized mouse before imaging. Animals were placed on a transparent sheet in the supine, prone, or lateral position. Images were acquired with a Kodak IS2000MM image station (Rochester, NY) with a 625/20 band-pass excitation filter, 700WA/35 band-pass emission filter, and 150 W quartz. Halogen lamp light source was set to maximum. Images were captured with a CCD camera set at F stop = 0, FOV = 150, and FP = 0. Data was collected at different time points and analyzed using the Kodak ID 3.6 software by drawing a region of interest (ROI) on the imaged mouse. For ex vivo imaging, the mice were euthanized, and their organs were excised for imaging. For bioluminescence imaging, 100 μL of 10 mg/mL luciferine per 10 g body weight was injected peritoneally 20 min prior to imaging with the light source off. Three mice were used for all the in vivo and ex vivo imaging experiments, and representative data were presented.

### Data processing and statistics

Histogram analysis for determination of MFI was conducted for flow cytometry data. The IC_50_ of peptides was calculated using GraphPad Prism 5. For determination of tumor contrast, we calculated MFIs of the tumor area and of the normal tissue area by means of the ROI function using Kodak 1D image analysis software (Kodak). All of the data are shown as mean ± SD in *n* independent measurements.

## Results

### Design, synthesis, and screening of focused OBOC libraries

**Fig. 1:**
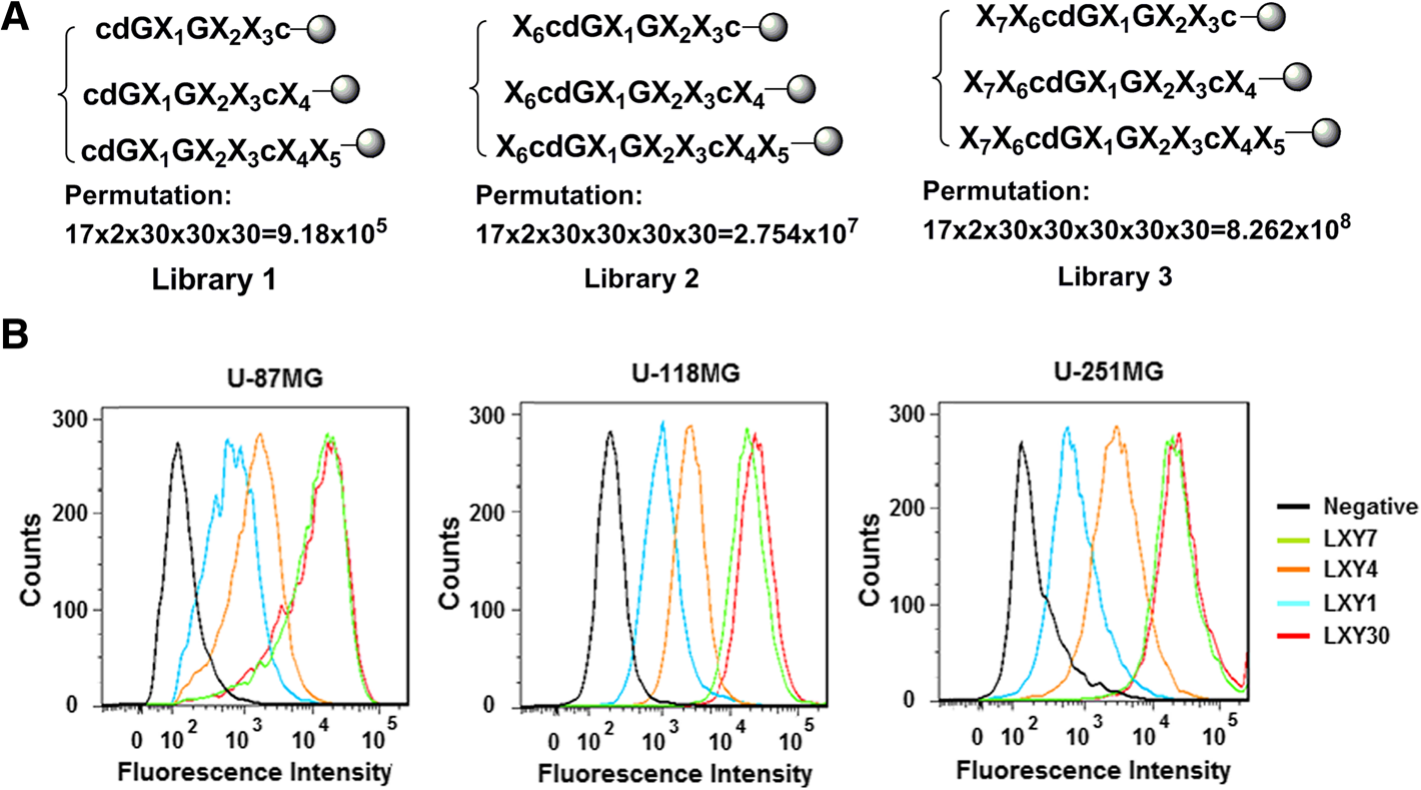
Structures of three focused OBOC libraries and binding affinity of various peptide ligands for α3β1 integrin-expressing glioblastoma cells by flow cytometry. The focused OBOC libraries were designed and synthesized (**a**). Three glioblastoma cell lines U-87MG, U-118MG, and U-251MG were incubated with biotinylated LXY1 (*blue curve*), LXY4 (*orange curve*), LXY7 (*green curve*), LXY30 (*red curve*), or negative control (*black curve*) and then analyzed with flow cytometry (**b**)

Three focused OBOC libraries were synthesized on TentaGel resin beads using a split-mix synthetic approach [19], and the structures are shown in Fig. 1a. These focused OBOC libraries were designed by fixing the motif cdG-G--c, and extending 1–2 random amino acids at the C-terminus alone (library 1) or together with further modification at the N-terminus of library 1 (libraries 2 and 3, respectively) with 30 different amino acids, including l-, d-, and unnatural amino acids (Additional file 1: Figure S1). This approach enabled us to probe for additional necessary residues adjacent to the motif of the ligand, improving the binding affinity of LXY1 to α3β1 integrin and improving peptide stability. Seventeen hydrophobic amino acids were used at X_1_ position (Additional file 1: Figure S2). An “on-bead” whole-cell binding assay with U-87MG cells was used to screen the libraries. Thirteen peptide beads from library 1 were found to bind strongly to U-87MG cells, and the peptides were decoded as shown in Table 1 with a modified microsequencing method [17]. Interestingly, LXY1 is among these 13 ligands. Five of these 13 peptide ligands have one added l-amino acid at the C-terminus of LXY1, two of which have an l-arginine. In addition, among the five ligands, four have side chains with hydrogen bond donor property that might form an additional hydrogen bond in the binding pocket. Beads displayed with peptides from libraries 2 and 3, extension of additional residues at the N-terminus of library 1, did not exhibit any stronger binding to U-87MG cells.

**Table 1:** Sequences of 13 peptides on beads bound to U-87MG cells from screening focused OBOC library 1 (cyclic cdGX_1_GX_2_X_3_c and cdGX_1_GX_2_X_3_cX_4_)

No.	X_1_	X_2_	X_3_	X_4_
1	HoPhe	Hyp	Phe(4-Me)	
2 (LXY21)	Phe(3,5-diF)	Hyp	Y	
3 (LXY23)	Phe(3,5-diF)	Hyp	Orn	W
4	L	P	R	
5	Phe(4-Me)	Hyp	N	
6 (LXY8)	L	Hyp	N	D
7	Nle	Hyp	W	R
8 (LXY20)	L	Hyp	D-Thi	
9 (LXY22)	L	Hyp	R	
10 (LXY10)	L	Hyp	D	G
11 (LXY19)	Phe(3,5-diF)	Hyp	S	
12 (LXY1)	L	Hyp	N	
13	L	Hyp	S	R

Natural amino acids are designated by the standard single letter code

Other abbreviations: *HoPhe* homophenylalanine, *Hyp* hydroxyproline, *Phe(4-Me)* 4-methylphenylalanine, *Phe(3,5-diF)* 3,5-difluorophenylalanine, *Orn* ornithine, *Nle* norleucine, *D-Thi* D-3-(2-thienyl)alanine

### Optimization of LXY1 using medicinal chemistry

Based on previously reported SAR information on LXY1 [16, 22, 23] and the aforementioned results from screening of focused OBOC libraries, we designed and synthesized 28 new peptide analogs of LXY1 by site-specific modification of individual amino acids (Table 2). A competition binding assay was used to determine the RBI of these peptides to U-87MG cells. We identified eight peptide ligands, i.e., LXY5, LXY7, LXY9, LXY14, LXY15, LXY29, LXY30, and LXY36, which have much stronger binding affinity against α3β1 integrin-expressing U-87MG glioblastoma cells compared to the lead ligand LXY1 and LXY4. Table 2 summarizes the sequences and RBI of these ligands.

**Table 2:** Peptide sequences of LXY1 derivatives (cyclic cdGX_1_G-Hyp-X_3_cX_4_) and their binding affinities against U-87MG cells

	X_1_	X_2_	X_3_	X_4_	Relative binding index	IC_50_ (μM)
LXY1	L	G	N		1.00 ± 0.05	3.5 ± 0.4
LXY4	Phe(3,5-diF)	G	N		1.36 ± 0.11	0.41 ± 0.03
LXY5	Phe(3,4,5-triF)	G	N	D-T	1.45 ± 0.12	ND
LXY6	Phe(3,5-diF)	G	N	W	1.17 ± 0.08	ND
LXY7	Phe(3,4,5-triF)	G	N	R	1.62 ± 0.10	0.13 ± 0.012
LXY8	L	G	N	D	0.22 ± 0.03	ND
LXY9	Phe(3,5-diF)	G	N	D-S	1.44 ± 0.11	ND
LXY10	L	G	D	G	0.64 ± 0.05	ND
LXY11	Phe(3,4-diF)	G	N	D-K	1.01 ± 0.10	ND
LXY12	Phe(3,4-diF)	G	N	S	0.47 ± 0.05	ND
LXY13	Phe(3,4,5-triF)	G	N	D	0.57 ± 0.03	ND
LXY14	Phe(3,4,5-triF)	G	N	D-S	1.35 ± 0.09	ND
LXY15	Phe(3,5-diF)	G	N	D-K	1.31 ± 0.08	ND
LXY16	Phe(3,5-diF)	G	N	S	0.61 ± 0.05	ND
LXY17	Phe(3,4-diF)	G	N	G	0.65 ± 0.06	ND
LXY18	Phe(3,4-diF)	G	N	W	0.01 ± 0.008	ND
LXY19	Phe(3,5-diF)	G	S		0.84 ± 0.07	ND
LXY20	L	G	D-Thi		0.15 ± 0.02	ND
LXY21	Phe(3,5-diF)	G	Y		0.62 ± 0.04	ND
LXY22	L	G	R		0.93 ± 0.1	ND
LXY23	L	G	Orn	W	0.7 ± 0.06	ND
LXY29	Phe(3,4-diF)	G	N	R	1.59 ± 0.09	ND
LXY30	Phe(3,5-diF)	G	N	R	1.68 ± 0.10	0.08 ± 0.01
LXY32	L	G	N	R	0.94 ± 0.07	ND
LXY33	OLeu^1^	G	N	R	0.81 ± 0.1	ND
LXY34	*N*- [(3,4,5-triF)benzyl]Gly^2^	G	N	R	0.68 ± 0.06	ND
LXY36	Phe(3,4,5-triF)	G	N	D-R	1.39 ± 0.10	ND
LXY37	L	G	N	D-R	0.67 ± 0.06	ND
LXY38	Phe(3,5-diF)	G	N	D-R	0.69 ± 0.05	ND
LXY39	Phe(3,5-diF)	Aoa^3^	N	D-R	1.09 ± 0.09	ND

1: 2: 3:

*Phe(3,4,5-triF)* 3,4,5-trifluorophenylalanine, *Aad* α-aminohexanedioic acid, *Aoa* aminooxy acetic acid, *ND* not determined

Relative binding index (RBI) of each peptide was determined as described below. Peptide at 0.4 μM was used to compete for the binding of 0.5 μM LXY1-biotin to U-87MG cells, followed by incubation with streptavidin-PE before analyzing by flow cytometry. Mean fluorescence intensity (MFI) was used as a quantitative measurement. The RBI for each sample was determined by the following formula: (MFI of positive control − MFI of the sample)/(MFI of positive control − MFI of LXY1). Positive control: 0.5 μM of LXY1-biotin. The peptide with greater RBI has higher binding affinity. When IC_50_ of the peptide was determined, the tested peptide with a series of concentration competed with 0.5 μM LXY1-biotin binding to U-87MG cells

### Characterization of LXY30 binding to glioblastoma cells

**Fig. 2:**
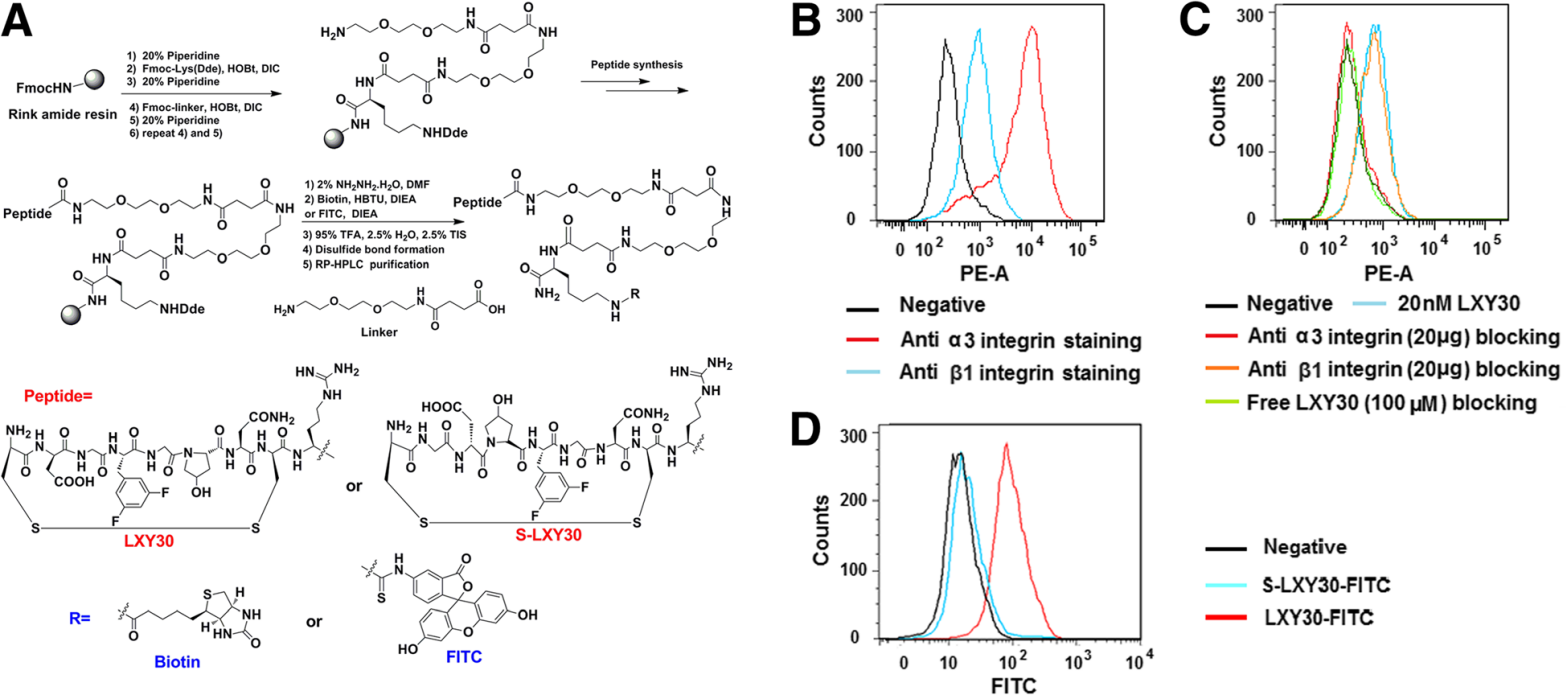
Synthesis and chemical structures of several peptide conjugates and binding specificity of LXY30 to U-87MG cells. The synthesis and chemical structures of peptide conjugates LXY30-biotin, LXY30-FITC, and scrambled-LXY30-FITC (S-LXY30-FITC) used for in vitro and in vivo characterization, as well as imaging studies were shown (**a**). U-87MG cells demonstrated obvious α3β1 integrin expression (**b**). The binding of 20 nM LXY30-biotin (*blue curve*) to U-87MG cells could be prevented by pre-incubating the cells with 20 μg anti-α3 antibody (*red curve*), or 100 μM free LXY30 (*green curve*), but not 20 μg anti-β1 antibody (*orange curve*) for 30 min before adding the LXY30-biotin (**c**). Compared with the negative control (*black curve*), LXY30-FITC (*red curve*), but not S-LXY30-FITC (*blue curve*), binds specifically to α3β1 integrin-expressing glioblastoma U-87MG cells (**d**)

**Fig. 3:**
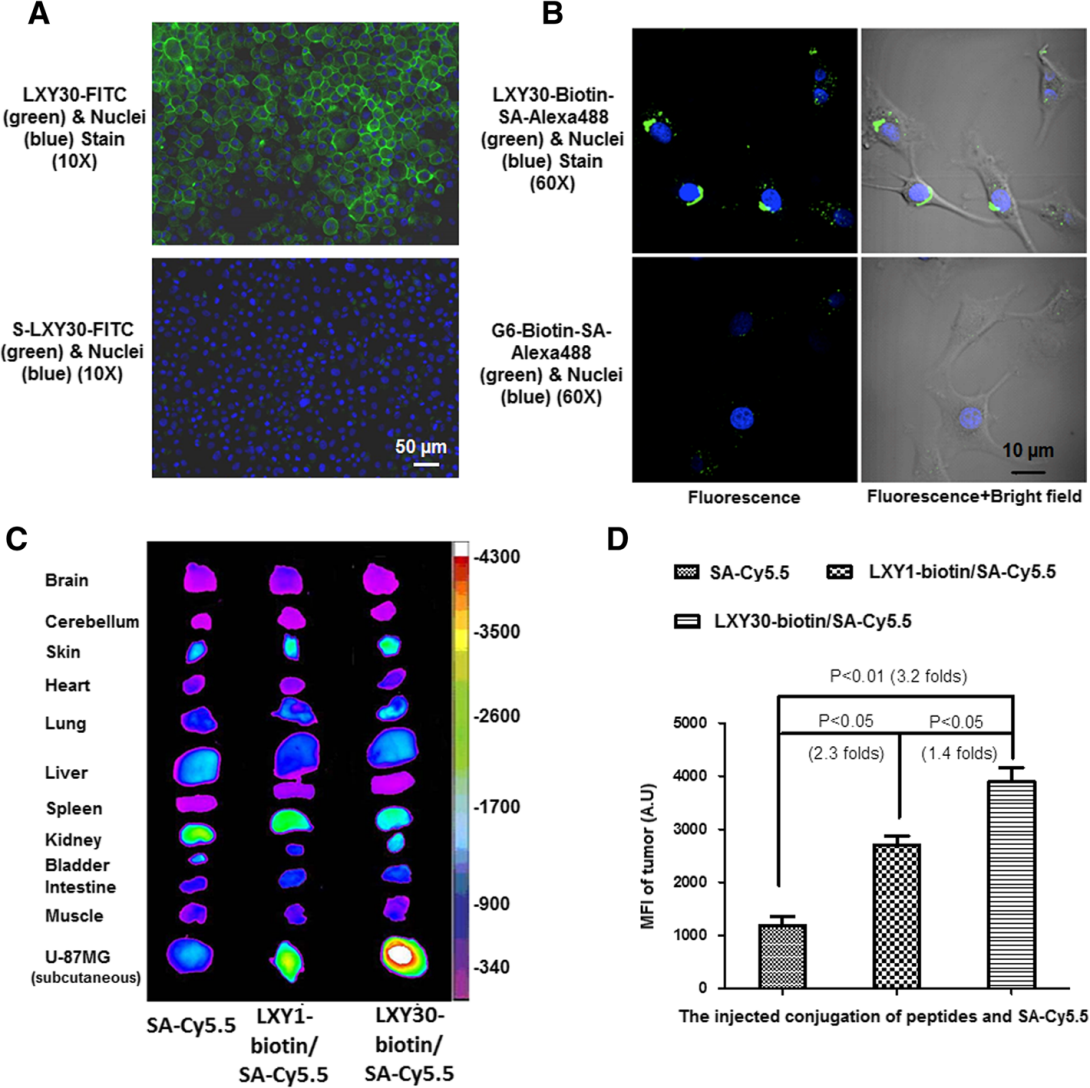
Microscopy analysis of LXY30 on U-87MG cells and optical images of LXY30 in subcutaneous α3β1 integrin-expressing glioblastoma U-87MG tumors. U-87MG cells were incubated with LXY30-FITC or S-LXY30-FITC and then spun down onto slides for visualization by immunofluorescence microscopy. LXY30-FITC, but not S-LXY30-FITC, binds to the α3β1 integrin on the surface of glioblastoma U-87MG cells within 30 min of incubation. DAPI (diamidino-2-phenylindole) was used for the nuclear staining (*blue*). *Scale bar*: 50 μm (**a**). LXY30 also indicated accumulation inside U-87MG when cells were incubated for 2 h with LXY30-biotin conjugated with streptavidin (SA)-Alexa 488 under a confocal fluorescence microscope. The G6 peptide (GGGGGG) was used as negative control. *Scale bar*: 10 μm (**b**). LXY1-biotin/SA-Cy5.5 and LXY30-biotin/SA-Cy5.5 were injected into nude mice bearing subcutaneous U-87MG xenograft tumors. The mean fluorescence intensity of LXY30 in the region of interest at each xenograft tumor was quantified and compared to those of LXY1 and SA-Cy5.5 dye alone. LXY30 delivers more SA-Cy5.5 dye to the xenograft tumor compared to that of LXY1 (**c**, **d**)

We compared the binding ability of a few lead peptides to inhibit the binding of 0.5 μM biotinylated LXY1 to U-87MG cells. LXY30 has a 44-fold higher binding affinity to U-87MG cells as compared to LXY1, i.e., the IC_50_ of LXY30 and LXY1 is 0.08 and 3.5 μM, respectively (Table 2). We also tested the binding profile of the four peptide ligands (LXY1, LXY4, LXY7, and LXY30) against two other glioblastoma cell lines, U-118MG and U-251MG. Among the four peptide ligands, LXY30 has the highest binding affinity for all three glioblastoma cell lines (Fig. 1b) and was selected for further biological characterization in this report. To facilitate further characterization, we synthesized LXY30-biotin and LXY30-FITC conjugates and scrambled LXY30 [cGd-Hyp-Phe(3,5-diF)-GNcR] conjugated with FITC (S-LXY30-FITC) using the approach shown in Fig. 2a. To determine the binding specificity of LXY30, we incubated U-87MG cells which express α3β1 integrin (Fig. 2b) with 20 nM LXY30-biotin (blue curve) (Fig. 2c) or negative control (10 % fetal bovine serum in 1× PBS, black curve). The binding of LXY30-biotin to U-87MG cells could be inhibited or prevented by pre-incubating the cells with 20 μg of anti-α3 antibody (red curve), or 100 μM of free LXY30 (green curve), but not 20 μg of anti-β1 antibody (orange curve) for 30 min before adding the 20 nM of LXY30-biotin. Thus, we have shown that peptide LXY30 bound to the α3 subunit of α3β1 integrin (Fig. 2c). To eliminate the possibility of nonspecific binding from the streptavidin-biotin complex, fluorophore FITC was directly conjugated with LXY30 or scrambled LXY30 (Fig. 2a) and used for binding. LXY30-FITC and S-LXY30-FITC were incubated with U-87MG cells, and the binding affinity was determined by flow cytometry analysis. Compared with the negative control (black curve), LXY30-FITC (red curve), but not S-LXY30-FITC (blue curve), binds to α3β1 integrin-expressing glioblastoma U-87MG cells (Fig. 2d). This specific binding of LXY30-FITC was further confirmed by the fluorescence microscopy (Fig. 3a) within 30 min of incubation. Furthermore, LXY30-biotin/streptavidin (SA)-Alexa488 has significant accumulation in the cytoplasm of U-87MG cells compared with control GGGGGG (G6)-biotin/SA-Alexa488 (Fig. 3b) after 2 h of incubation. It indicates that LXY30 not only targets the surface of tumor cells but also internalizes into the cells given longer time of interaction. The results support the use of LXY30 as a drug delivery carrier.

### In vivo biodistribution study of LXY30 in nude mice bearing subcutaneous glioblastoma U-87MG tumors using near infrared fluorescence (NIRF) optical imaging

To compare the in vivo biodistribution of LXY30 with LXY1, LXY30-biotin/SA-Cy5.5 complex, LXY1-biotin/SA-Cy5.5 complex, and SA-Cy5.5 were each injected into nude mice bearing subcutaneous U-87MG xenograft tumors (Fig. 3c). The MFI of LXY30 in the ROI of each xenograft tumor was quantified and compared to those of LXY1 and SA-Cy5.5 dye alone. Figure 3d illustrates that while LXY1 alone could target 2.3-fold more SA-Cy5.5 dye to the xenograft tumor, LXY30 facilitates an additional increase of 1.4-fold to the tumor compared to LXY1.

### LXY30 targets the orthotopic xenograft tumors of U-87MG cells

**Fig. 4:**
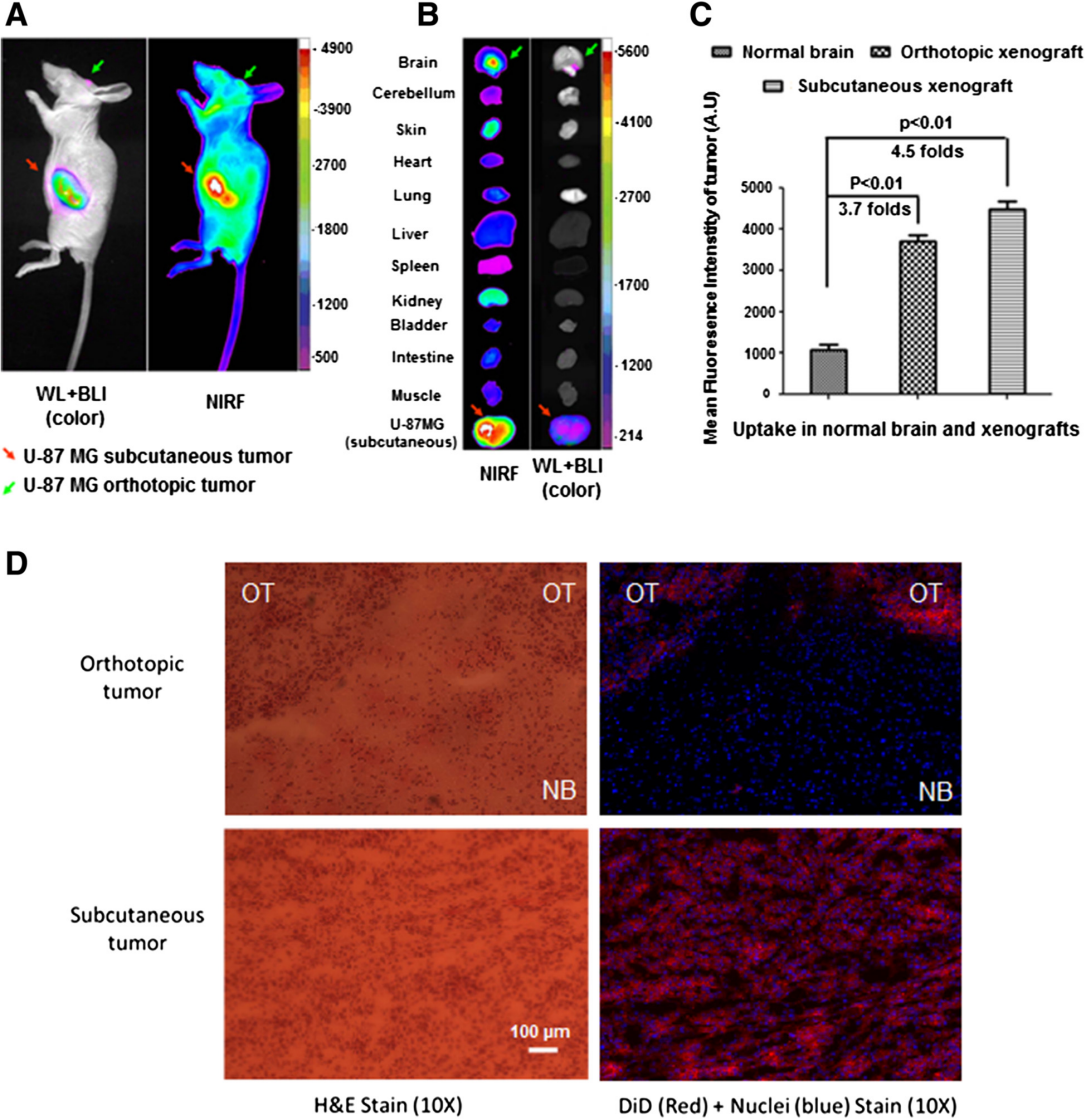
Integrin ligand LXY30 targets the conjugated SA-Cy5.5 dye to the subcutaneous and orthotopic xenograft tumors of α3β1 integrin-expressing U-87MG cells. U-87MG cells were stably transfected with luciferase and engrafted in the nude mice to generate both subcutaneous and orthotopic xenograft tumors. The mice were subjected to in vivo (**a**) and ex vivo (**b**) bioluminescence imaging (BLI) and optical NIRF imaging after LXY30-biotin/SA-Cy5.5 was injected. Both imaging approaches confirmed the uptake of LXY30-biotin/SA-Cy5.5 in the orthotopic xenograft tumors as well as the subcutaneous xenograft tumors of U-87MG cells. **c** The quantification of NIRF images indicated that both subcutaneous and orthotopic xenografts had higher uptakes of LXY30-biotin/SA-Cy5.5 compared to that of the normal brain. **d** Light microscopy of H&E staining and fluorescence microscopy of frozen sections of orthotopic and subcutaneous U-87MG xenograft tumors. LXY30-biotin/SA-Cy5.5 only accumulated in the subcutaneous (*lower panel*) and orthotopic U-87MG xenograft tumor cells, but not in adjacent normal tissue in the brain (*upper panel*). *OT* orthotopic tumor, *NB* normal brain. *Scale bar*: 100 μm

LXY30 is considerably stable in plasma as demonstrated in an in vitro stability study in 90 % human plasma (Additional file 1: Method S2, Figure S3). The half-life of LXY30 in human plasma is calculated to be 16.0 days so that LXY30 is a suitable optical imaging agent for in vivo administration. The in vivo tumor-targeting effect of LXY30 was evaluated in an orthotopic mouse model by optical imaging. Briefly, U-87MG cells were stably transfected with luciferase and engrafted in the nude mice to generate both subcutaneous and orthotopic xenograft tumors. Six hours after LXY30-biotin/SA-Cy5.5 complex was injected, the mice were subjected to both in vivo (Fig. 4a) and ex vivo (Fig. 4b) bioluminescence imaging (BLI) and optical NIRF imaging. Both imaging approaches confirmed the accumulation of LXY30-biotin/SA-Cy5.5 complex in the orthotopic xenograft tumors in addition to the subcutaneous xenograft tumors derived from implanted α3β1 integrin-expressing U-87MG cells. We quantified the NIRF images and found that both subcutaneous and orthotopic xenografts had significant higher uptakes of LXY30-biotin/SA-Cy5.5 compared to that of the normal brain (Fig. 4c). We further visualized the uptakes of LXY30 in the orthotopic and subcutaneous U-87MG xenograft tumors, which showed expression of α3 integrin (Additional file 1: Method S3, Figure S4), by hematoxylin and eosin (H&E) stain under light (left panel) and fluorescence (right panel) microscopy. We also found that LXY30-biotin/SA-Cy5.5 only accumulated in the subcutaneous U-87MG xenograft tumor cells (lower panel) and orthotopic U-87MG xenograft tumor cells but not adjacent normal tissue in the brain (upper panel) (Fig. 4d).

### LXY30 binds to various human breast and lung cancer cell lines

As α3β1 integrin is overexpressed in several cancer types including breast and lung cancers, we evaluated the binding of LXY30 against a panel of breast and lung cancer cell lines by flow cytometry. We found that LXY30 bound to different tumor cell lines with a wide range of variable affinity, which was well correlated with the messenger RNA (mRNA) expression level of α3β1 integrin on these cell lines [25] (Additional file 1: Table S2, Figure S5, and Method S4).

## Discussion

Among the several available approaches, the biological library (such as phage display library) and OBOC combinatorial library are the two most commonly used methods to identify cancer-targeting peptide ligands [26]. Yet unlike the biological combinatorial library approach, which is generally limited to l-amino acids, ligands identified from OBOC libraries can contain unnatural amino acids (e.g., d- and β-amino acids), organic moieties, and/or constrained conformation which improve the stability of the ligands against proteolysis. Therefore, it is a rapid, high-throughput, and cost-effective approach to identify integrin-targeting ligands. Using the OBOC approach, we previously discovered three cyclic peptide ligands that target α3β1 in U-87MG cells: LXY1, LXY3, and LXY4. The order of in vitro binding affinity to U-87MG cells is LXY4>LXY3>LXY1. However, LXY3 or LXY4 did not show improvement in tumor targeting over LXY1 in optical imaging studies on a U-87MG glioblastoma xenograft model (data not shown). We hypothesized that further optimization of ligand with higher affinity would be able to improve in vivo tumor-targeting capacity.

In this report, we used a combination of the focused OBOC library and traditional medicinal chemistry approaches to rapidly identify LXY30, an improved and potent ligand against the α3β1 integrin. First, we synthesized three focused OBOC libraries designed for targeting α3β1 integrin. Many d- and unnatural amino acids were used as building blocks to increase in vivo stability. In order to minimize the time and cost of synthesis and screening of OBOC libraries, all three OBOC libraries L1–L3 contain three sub-libraries that have 0–2 amino acids at the C-terminal of LXY1. In theory, we should have been able to identify LXY30 from the screening of library L1, but we failed to pick it up. The reason for this is that the actual number of screened beads (200-μL beads, ~1.5 × 10^5^) is lower than the theoretical permutation (9.18 × 10^5^). In order to cover all possible ligands, a tenfold high number of permutation beads would have to be screened which is neither practical nor necessary. Nevertheless, we had obtained very useful SAR information from screening the three OBOC libraries (Fig. 1 and Table 1): No additional amino acid is needed at the N-terminus of LXY1, but a hydrogen donor residue (or a polar residue) at the C-terminus might improve binding since it can provide additional interaction with adjacent binding pocket. In addition, hydroxyproline (Hyp) is more favorable (12 out of 13) than l-proline (P) for the X_2_ position because its hydroxyl group can form an extra hydrogen bond with α3β1 integrin. Based on this SAR information, 28 new analogs of LXY1 were designed with majority having a hydrogen bond-forming (donor or acceptor) amino acid at the C-terminus. Those compounds were quickly synthesized using standard solid-phase peptide synthesis approach and tested using an in vitro cell binding assay. LXY30 was found to be most potent (Table 2). At the X_1_ position, the order of ligand binding affinity is Phe(3,5-diF) > Phe(3,4,5-triF) > Phe(3,4-diF) > L, which is consistent with our previous finding indicating the finding was not obtained by chance [22]. Introduction of l-arginine, a positively charged amino acid, at the X_4_ position of the C-terminus resulted in an increase in binding affinity (LXY30 vs LXY4). In contrast, a negatively charged amino acid at the X_4_ position decreased binding (LXY13 vs LXY14 and LXY8 vs LXY1). However, ligands with d-arginine at the X_4_ position have weaker binding than their l-arginine counterpart (LXY38 vs LXY30, LXY36 vs LXY7), indicating that the conformation of arginine is also important for the binding. Insertion of an oxygen atom in the peptide chain (LXY33 and LXY39) resulted in reduced binding, implying that the size or polarity of the ring should not be changed. Replacement of the amino acid with *N*-substituted glycine led to a significant loss of binding (LXY7 vs LXY34), indicating that the side chain appended to the α-carbon is very important for the binding activity. The resulting peptide, LXY30, not only had improved in vitro binding to all three tested glioblastoma cells (Fig. 1) but also demonstrated better in vivo tumor targeting than LXY1 (Fig. 3). We further tested the binding of LXY30 to a panel of lung and breast cancer cell lines and found that LXY30 bound to different tumor cell lines with a wide range of variable affinity (Additional file 1: Table S2 and Figure S3). It can be explained by the heterogeneous expression level of α3β1 integrin on these cell lines verified by a recently published report [25], in which the trend of α3β1 integrin mRNA expression levels on these cell lines is consistent with their LXY30 binding affinities (Additional file 1: Table S2).

Our study has several translational potentials. First, overexpression of α3β1 integrin has been shown in multiple, aggressive tumor types, including basal-like breast cancer cells and lung cancer cells [9, 27]. Recent studies suggest that α3β1 integrin-mediated signaling plays a critical role in the initiation and/or growth of mammary tumors [28]. Overexpression of α3β1 integrin is associated with spontaneous metastasis of breast [14] and lung cancer cells to the brain [13] and mediates the resistance of HER2+ breast cancer cells to cancer therapy [29]. Second, the internalization property of LXY30 on α3β1 integrin-expressing cancer cells could be used to facilitate delivery of conventional chemotherapeutic agents, target-specific agents, siRNAs, and microRNAs into tumor cells, either through direct conjugation or by encapsulation inside LXY30-decorated nanocarriers. Third, there is a largely unmet need to improve drug delivery to refractory, metastatic tumors while sparing the normal cells that have been exposed to accumulative dose of cancer therapeutics. Finally, while malignant gliomas are the most prevalent type of primary brain tumor in adults, central nervous system (CNS) metastases from epithelial tumors are much more common than primary brain tumors [30]. The incidence of CNS metastases has been rising in recent years due to early detection with imaging and improved systemic therapy (especially oncogene-driven molecularly targeted therapy) for extra-CNS tumors [31, 32]. CNS metastases may occur in 20–40 % of patients with cancer, of which 60–75 % are symptomatic. In adults, lung cancer is the number one primary cancer that is most likely (>50 %) to metastasize to the brain, followed by breast (15–25 %) and skin (melanoma, 5–20 %) cancer [30, 33]. Patients with CNS metastases often have short survival and significant mental and physical debilitations that create extra burdens on both the patient and family members. This represents another unmet clinical need to improve the diagnosis and treatment for CNS metastasis. The fact that LXY30-biotin/streptavidin-Cy5.5 complex with over 80,000 Da can target intracranial implanted xenografts (Fig. 4) indicates that LXY30 is an excellent cancer-specific ligand for guiding drug delivery to primary or metastatic tumors in the brain.

## Conclusions

In conclusion, we have discovered and characterized LXY30 as a highly potent and selective peptide ligand for targeting live α3β1 integrin-expressing tumor cells. Compared to the parent LXY1 peptide, LXY30 binds to the α3 subunit of α3β1 integrin on the surface of glioblastoma cells with a 44-fold higher binding affinity and enters into the tumor cells. Furthermore, LXY30 binds to the surface of a panel of basal-like breast and metastatic NSCLC cell lines with various affinities. Therefore, LXY30 is a tumor-specific peptide ligand that has great translational potential for systemic and intracranial delivery of imaging agents and/or cancer therapeutics to α3β1 integrin-expressing human tumors.

## Additional file

Additional file 1: Tables S1–S3, Figures S1–S5.
**Methods S1–S4.** (DOCX 1572 kb)

## Abbreviations

BLI: bioluminescence imaging
CNS: central nervous system
Da: Daltons
DAPI: 4′,6-diamidino-2-phenylindole
DIC: 1,3-diisopropylcarbodiimide
ECM: cell-to-extracellular matrix
EDTA: ethylenediaminetetraacetic acid
FBS: fetal bovine serum
HOBt: 1-hydroxybenzotriazole
Hyp: hydroxyproline
IACUC: Institutional Animal Care and Use Committee
lower case “c”: d-cysteine
MFI: mean fluorescence intensity
NSCLC: non-small cell lung cancer
OBOC: one-bead one-compound
PBS: phosphate-buffered saline
PE: phycoerythrin
RBI: relative binding index
ROI: region of interest
SA: streptavidin
SAR: structure-activity relationship
TFA: trifluoroacetic acid
TIS: triisopropylsilane
