# Supplementary material for: Discovery and characterization of a high-affinity and high-specificity peptide ligand LXY30 for in vivo targeting of α3 integrin-expressing human tumors
Source: EJNMMI Res. 2016 Feb 27;6:18. doi: 10.1186/s13550-016-0165-z (PMC4769701; doi:10.1186/s13550-016-0165-z)
Supplement: Additional file 1: Tables S1–S3, Figures S1–S5. — Methods S1–S4. (DOCX 1572 kb) [file 13550_2016_165_MOESM1_ESM.docx]

**Discovery and characterization of a high affinity and specificity peptide ligand LXY30 for *in vivo* targeting of α3 integrin-expressing human tumors**

**Wenwu Xiao^†^, Tianhong Li^‡,§,ǁ^, Fernanda C. Bononi^†^, Diana Lac^†^, Ivy A. Kekessie^†^, Yanlei Liu^#^, Eduardo Sanchez^†^, Anisha Mazloom^†^, Ai-hong Ma^†^, Jia Lin^†^, Jimmy Tran^†^,** **Kevin Yang^‡@^, Kit S. Lam^†,‡,§^, and Ruiwu Liu*^,†,§^**

†. Department of Biochemistry and Molecular Medicine, University of California Davis, Sacramento, California 95817, USA

‡. Division of Hematology/Oncology, Department of Internal Medicine, University of California Davis, Sacramento, California 95817, USA

§. University of California Davis Comprehensive Cancer Center, Sacramento, California 95817, USA

ǁ. Veterans Affairs Northern California Health Care System, Mather, CA 95655, USA

#. Department of Pathology and Laboratory Medicine, University of California Davis, Sacramento, California 95817, USA

@. Current address: the College of the University of Chicago, Chicago, IL 60602, USA

**Table S1. HR ESI-MS data of resynthesized peptide ligands**

| **Peptide** | **Molecular Formula** | **Calculated MW*** | **Measured MW*** |
| --- | --- | --- | --- |
| LXY5 | [C_36_H_49_F_3_N_11_O_14_S_2_ + H]^+^ | 980.2854 | 980.2870 |
| LXY6 | [C_43_H_53_F_2_N_12_O_13_S_2_ + H]^+^ | 1047.3265 | 1047.3347 |
| LXY7 | [C_38_H_54_F_3_N_14_O_13_S_2_ + H]^+^ | 1035.3388 | 1035.3391 |
| LXY8 | [C_33_H_52_N_11_O_15_S_2_ + H]^+^ | 906.3086 | 906.3152 |
| LXY9 | [C_35_H_48_F_2_N_11_O_14_S_2_ + H]^+^ | 948.2792 | 948.2872 |
| LXY10 | [C_31_H_49_N_10_O_14_S_2_ + H]^+^ | 849.2871 | 849.2907 |
| LXY11 | [C_38_H_55_F_2_N_12_O_13_S_2_ + H]^+^ | 989.3421 | 989.3405 |
| LXY12 | [C_35_H_48_F_2_N_11_O_14_S_2_ + H]^+^ | 948.2792 | 948.2824 |
| LXY13 | [C_36_H_47_F_3_N_11_O_15_S_2_ + H]^+^ | 994.2647 | 994.2721 |
| LXY14 | [C_35_H_47_F_3_N_11_O_14_S_2_ + H]^+^ | 966.2697 | 966.2785 |
| LXY15 | [C_38_H_55_F_2_N_12_O_13_S_2_ + H]^+^ | 989.3421 | 989.3494 |
| LXY16 | [C_35_H_48_F_2_N_11_O_14_S_2_ + H]^+^ | 948.2792 | 948.2891 |
| LXY17 | [C_34_H_46_F_2_N_11_O_13_S_2_ + H]^+^ | 918.2686 | 918.9217 |
| LXY18 | [C_43_H_53_F_2_N_12_O_13_S_2_ + H]^+^ | 1047.3265 | 1047.3374 |
| LXY19 | [C_31_H_42_F_2_N_9_O_12_S_2_ + H]^+^ | 834.2362 | 834.2401 |
| LXY20 | [C_32_H_48_N_9_O_11_S_3_ + H]^+^ | 830.2635 | 830.2713 |
| LXY21 | [C_37_H_46_F_2_N_9_O_12_S_2_ + H]^+^ | 910.2675 | 910.2762 |
| LXY22 | [C_31_H_53_N_12_O_11_S_2_ + H]^+^ | 833.3398 | 833.3464 |
| LXY23 | [C_41_H_61_N_12_O_12_S_2_ + H]^+^ | 977.3973 | 977.4066 |
| LXY29 | [C_38_H_55_F_2_N_14_O_13_S_2_ + H]^+^ | 1017.3483 | 1017.3534 |
| LXY30 | [C_38_H_55_F_2_N_14_O_13_S_2_ + H]^+^ | 1017.3483 | 1017.3498 |
| LXY32 | [C_35_H_59_N_14_O_13_S_2_ + H]^+^ | 947.3827 | 947.3905 |
| LXY33 | [C_35_H_59_N_14_O_14_S_2_ + H]^+^ | 963.3777 | 963.3853 |
| LXY34 | [C_38_H_54_F_3_N_14_O_13_S_2_ + H]^+^ | 1035.3388 | 1035.3484 |
| LXY36 | [C_38_H_54_F_3_N_14_O_13_S_2_ + H]^+^ | 1035.3388 | 1035.3482 |
| LXY37 | [C_35_H_59_N_14_O_13_S_2_ + H]^+^ | 947.3827 | 947.3915 |
| LXY38 | [C_38_H_55_F_2_N_14_O_13_S_2_ + H]^+^ | 1017.3483 | 1017.3577 |
| LXY39 | [C_38_H_55_F_2_N_14_O_14_S_2_ + H]^+^ | 1033.3432 | 1033.3525 |

Abbreviations: MW, molecular weight. *: dalton

**Figure S1. Thirty Fmoc-amino acids used at position X_3_-X_7_ in the three focused OBOC libraries**

**Figure S2. Seventeen Fmoc-amino acids used at position X_1_ in the three focused OBOC libraries**

**Figure S3. The *in vitro* stability of LXY30 in human plasma.** LXY30 was incubated with 90% human plasma from healthy donor at 37°C for up to 8 days. The peptide concentrations were determined by integration of the LXY30 peak (area) from reversed-phase [high-performance liquid chromatography](https://en.wikipedia.org/wiki/High-performance_liquid_chromatography) (RP-HPLC) chromatograms at UV 214 nm. The half-life of LXY30 in the plasma was calculated as 16.0 days using the formula *t*_1/2_=1/*k*_obs_**·**ln(2). Assays were performed in triplicate.


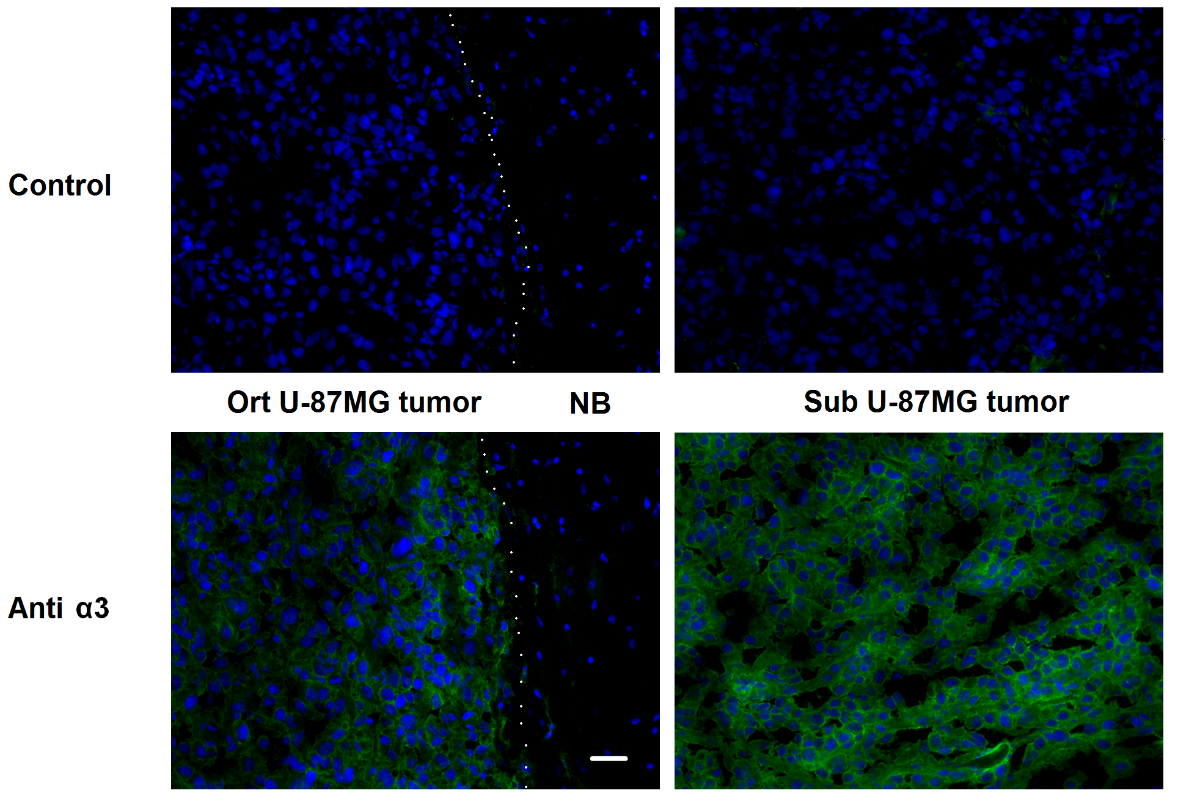


**Figure S4. Integrin α3 expression of U-87MG xenografts.** The orthotopic and subcutaneous U-87MG xenografts showed positive staining for integrin α3 expression. (Scale bar: 50 µm, Blue: DAPI staining, Green: anti-α3 integrin staining. NB: normal brain)

**
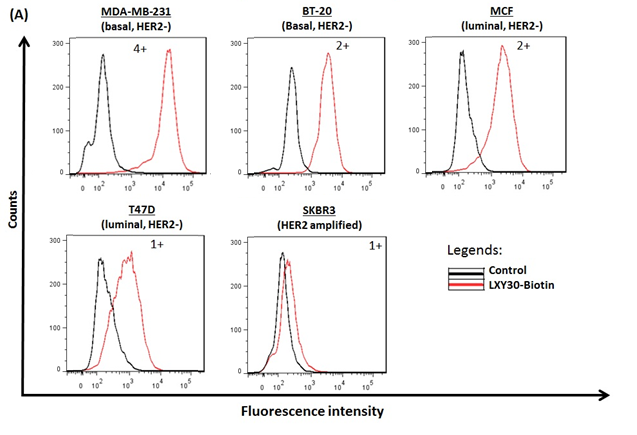
**


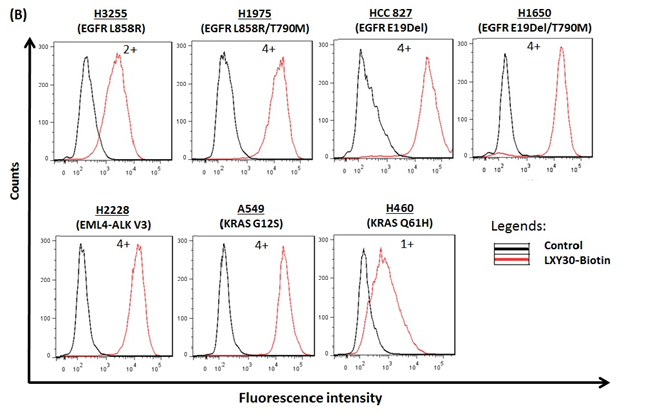


**Figure S5. Flow cytometry analysis of LXY30 binding to a panel of breast cancer (A) and lung cancer (B) cell lines (also see Table S2).** Black curves represent the negative controls. Red curves represent the cell binding after the addition of LXY30-Biotin.

**Table S2. Summary of LXY30 binding to various cancer cells by flow cytometry**

| **Cell Line** | **Tumor Type**  **(Tissue, subtype)** | **Clinically Relevant**  **Molecular Biomarkers** | **(MFI W/ LXY30)/**  **(MFI W/O LXY30)*** | **α3 integrin RNA level** |
| --- | --- | --- | --- | --- |
| MDA-MB 231 | Breast, basal like | ER-/PR-/HER2- | 4+ | 15.7 |
| BT-20 | Breast, basal like | ER-/PR-/HER2- | 2+ | 13.2 |
| MCF7 | Breast, luminal A | ER+/PR-/HER2- | 2+ | 11.5 |
| T47D | Breast, luminal A | ER+/PR+/HER2- | 1+ | 11.3 |
| SKBR3 | Breast, HER2 | ER-/PR-/HER2+ | 1+ | 11.1 |
| H3255 | Lung, adenocarcinoma | EGFR L858R | 2+ | NA |
| H1975 | Lung, adenocarcinoma | EGFR L858R/T790M | 4+ | 15.1 |
| HCC827 | Lung, adenocarcinoma | EGFR E19Del | 4+ | 15.3 |
| H1650 | Lung, bronchoalveolar | EGFR E19Del/T790M | 4+ | 14.7 |
| H2228 | Lung, adenocarcinoma | EML4-ALK V3a/b | 4+ | 15.7 |
| A549 | Lung, adenocarcinoma | KRAS G12S | 4+ | 14.9 |
| H460 | Lung, large cell | KRAS Q61H | 1+ | 11.3 |

*(MFI W/ LXY30)/ (MFI W/O LXY30): Mean Fluorescence Intensity of cells incubated with 1µM LXY30 divided by Mean Fluorescence Intensity of cells without incubation with LXY30.

Mean Fluorescence Intensity (MFI) was used as the quantitative measurement. The binding strength was grouped into five categories based on the value. “0”: 1 “1+”: 1.1~4.9 “2+”: 5~19.9 “3+”: 20~79.9 “4+”: > or = 80-fold

Abbreviations: ER, estrogen receptor; PR, progesterone receptor; HER, human EGF *(Epidermal Growth Factor)* receptor; EGFR, epidermal growth factor receptor; EML4, echinoderm microtubule-associated protein-like 4; ALK, anaplastic lymphoma kinase; WT, wild-type gene; E19Del, exon 19 deletion; KRAS, Kirsten rat sarcoma viral oncogene homolog.

**Method S1: ^1^H NMR of LXY30**

LXY30: ^1^H NMR (800 MHz, DMSO-*d_6_*) δ 8.94 (d, *J* - 8.3 Hz, 1H), 8.37 (s, 6H), 8.24 (t, *J* - 5.5 Hz, 1H), 8.21 (d, *J* - 9.2 Hz, 1H), 7.81 (d, *J* - 7.9 Hz, 1H), 7.77 (s, 1H), 7.64 (d, *J* - 4.8 Hz, 2H), 7.46 (s, 1H), 7.37 (s, 2H), 7.24 (s, 1H), 7.18 (s, 1H), 7.11 (t, *J* - 8.2 Hz, 3H), 7.10 – 7.07 (m, 1H), 7.05 (dd, *J* - 8.6, 2.2 Hz, 4H), 7.02 (s, 1H), 4.69 (dd, *J* - 13.5, 8.2 Hz, 1H), 4.66 – 4.60 (m, 2H), 4.44 (s, 1H), 4.39 (t, *J* - 8.2 Hz, 1H), 4.36 – 4.31 (m, 1H), 4.27 – 4.12 (m, 4H), 4.09 (dd, *J* - 13.8, 6.7 Hz, 1H), 3.91 (dd, *J* - 16.7, 7.7 Hz, 1H), 3.76 (d, *J* - 12.8 Hz, 1H), 3.25 (d, *J* - 10.2 Hz, 2H), 3.18 – 3.08 (m, 7H), 3.03 – 2.94 (m, 2H), 2.85 (dd, J - 15.1, 4.0 Hz, 1H), 2.81 – 2.75 (m, 2H), 2.72 (dd, *J* - 16.5, 8.4 Hz, 1H).

**Table S3. MALDI-TOF MS of synthesized peptide-conjugates**

| **Peptide-Conjugate** | **Molecular formula** | **Calculated MS*** | **Measured MS*** |
| --- | --- | --- | --- |
| LXY7-Biotin | [C_74_H_116_F_3_N_22_O_24_S_3_ + H]^+^ | 1849.76 | 1849.77 |
| LXY30-Biotin | [C_74_H_117_F_2_N_22_O_24_S_3_ + H]^+^ | 1831.77 | 1831.48 |
| S-LXY30-Biotin | [C_74_H_117_F_2_N_22_O_24_S_3_ + H]^+^ | 1831.77 | 1831.62 |
| LXY30-FITC | [C_85_H_114_F_2_N_21_O_27_S_3_ + H]^+^ | 1994.73 | 1994.81 |
| S-LXY30-FITC | [C_85_H_114_F_2_N_21_O_27_S_3_ + H]^+^ | 1994.73 | 1994.81 |

Abbreviations: MW, molecular weight. *: dalton

**Method S2: *In vitro* stability assay of LXY30 in human plasma**

Human plasma was separated from the peripheral blood collected in the EDTA-containing blood collection tube from healthy donors. One hundred microliters of LXY30 solution (2 mM in water) was mixed with 900 µL of plasma (final concentration of LXY30 was 200 µM) and incubated at 37°C under sterile conditions. Then 100 µL of the drug-plasma mixture was removed at appropriate intervals based on expected half-life and degradation was stopped by precipitation of the sample with 10 µL of acetonitrile (ACN)/trifluoroacetic acid (TFA) (50:50 v/v). The sampling intervals were 0.1 min, 0.25d, 0.5d, 1d, 2d, 3d, 4d, 6d and 8d. A control plasma sample without LXY30 but treated in the same manner was also analyzed. The precipitated plasma samples were centrifuged at 12,000 rpm at room temperature for 20 min. The supernatant was transferred to an empty tube and centrifuged for additional 10 min at 12,000 rpm. Fifty microliter of the resulting supernatant was injected to RP-HPLC to determine the concentration of LXY30. These assays were performed in triplicate.

RP-HPLC analysis was performed on the Waters 2996 HPLC system equipped with a Luna^®^ analytical column from Phenomenex (5 µm, C18, 100 A°, 250 x 4.6 mm). A linear gradient was run from 100% solution A (water/0.05% TFA) to 48% solution B (ACN/0.05% TFA) within 12 min with a flow rate at 1.0 mL/min. The relative concentrations of the remaining LXY30 were analyzed by the integration of the absorbance at 214 nm (area).

Half-life (*t*_1/2_) for LXY30 in the plasma solution was calculated from plots of the natural logarithm of the residual concentration against time using the formula *t*_1/2_=1/*k*_obs_**·**ln(2), where *k*_obs_ is the apparent first order rate constant for the observed degradation.

**Method S3: Xenografts stained with anti- α3 antibody**

Ten-micron cryosections of intracranial and subcutaneous U-87 MG xenografts were fixed in 4% paraformaldehyde at room temperature for 20 min. After washing with PBS, the sections were stained with mouse anti-α3 antibody (Chemicon International, Inc., Billerica, MA) at 1:250 for 1 h and followed by chicken anti-mouse IgG-Alexa488 (1:500) incubation for another hour. Then the sections were stained with DAPI and visualized under fluorescence microscope.

**Method S4: RNA sequencing data analysis**

RNA expression, copy number variation (CNV) and single-nucleotide polymorphism (SNP) data for the α3β1 integrin in 11 of the 12 breast and lung cancer cell lines in Table S2 were assessed from a recently published database of RNA sequencing and single-nucleotide polymorphism (SNP) array analysis of a large panel of human cancer cell lines [1]. In this database, RNA expression levels for *ITGA3* and *ITGAB1*, the subunits for the α3β1integrin heterodimer, were available as variance stabilized counts as outputted by the *DESeq*-based pipeline. Total RNA expression for the α3β1 integrin was calculated by summing the composite expression levels of *ITGA3* and *ITGAB1 for each* cancer cell line. CNV data were available for both genes as a transformed copy number value which normalizes the absolute copy number per gene by estimated ploidy for each cell line. Specifically, a copy number greater than 1 represents amplification over the normal ploidy number, and an absolute copy number smaller than -0.75 represents deletion. Finally, the data analysis provided a list of somatic mutations for each cancer cell line, called by comparison with a panel of normal genomes.

**Reference:**

1. [Klijn C](http://www.ncbi.nlm.nih.gov/pubmed/?term=Klijn%20C%5BAuthor%5D&cauthor=true&cauthor_uid=25485619), [Durinck S](http://www.ncbi.nlm.nih.gov/pubmed/?term=Durinck%20S%5BAuthor%5D&cauthor=true&cauthor_uid=25485619), [Stawiski EW](http://www.ncbi.nlm.nih.gov/pubmed/?term=Stawiski%20EW%5BAuthor%5D&cauthor=true&cauthor_uid=25485619), [Haverty PM](http://www.ncbi.nlm.nih.gov/pubmed/?term=Haverty%20PM%5BAuthor%5D&cauthor=true&cauthor_uid=25485619), [Jiang Z](http://www.ncbi.nlm.nih.gov/pubmed/?term=Jiang%20Z%5BAuthor%5D&cauthor=true&cauthor_uid=25485619), [Liu H](http://www.ncbi.nlm.nih.gov/pubmed/?term=Liu%20H%5BAuthor%5D&cauthor=true&cauthor_uid=25485619), [Degenhardt J](http://www.ncbi.nlm.nih.gov/pubmed/?term=Degenhardt%20J%5BAuthor%5D&cauthor=true&cauthor_uid=25485619), [Mayba O](http://www.ncbi.nlm.nih.gov/pubmed/?term=Mayba%20O%5BAuthor%5D&cauthor=true&cauthor_uid=25485619), [Gnad F](http://www.ncbi.nlm.nih.gov/pubmed/?term=Gnad%20F%5BAuthor%5D&cauthor=true&cauthor_uid=25485619), [Liu J](http://www.ncbi.nlm.nih.gov/pubmed/?term=Liu%20J%5BAuthor%5D&cauthor=true&cauthor_uid=25485619), [Pau G](http://www.ncbi.nlm.nih.gov/pubmed/?term=Pau%20G%5BAuthor%5D&cauthor=true&cauthor_uid=25485619), [Reeder J](http://www.ncbi.nlm.nih.gov/pubmed/?term=Reeder%20J%5BAuthor%5D&cauthor=true&cauthor_uid=25485619), [Cao Y](http://www.ncbi.nlm.nih.gov/pubmed/?term=Cao%20Y%5BAuthor%5D&cauthor=true&cauthor_uid=25485619), [Mukhyala K](http://www.ncbi.nlm.nih.gov/pubmed/?term=Mukhyala%20K%5BAuthor%5D&cauthor=true&cauthor_uid=25485619), [Selvaraj SK](http://www.ncbi.nlm.nih.gov/pubmed/?term=Selvaraj%20SK%5BAuthor%5D&cauthor=true&cauthor_uid=25485619), [Yu M](http://www.ncbi.nlm.nih.gov/pubmed/?term=Yu%20M%5BAuthor%5D&cauthor=true&cauthor_uid=25485619), [Zynda GJ](http://www.ncbi.nlm.nih.gov/pubmed/?term=Zynda%20GJ%5BAuthor%5D&cauthor=true&cauthor_uid=25485619), [Brauer MJ](http://www.ncbi.nlm.nih.gov/pubmed/?term=Brauer%20MJ%5BAuthor%5D&cauthor=true&cauthor_uid=25485619), [Wu TD](http://www.ncbi.nlm.nih.gov/pubmed/?term=Wu%20TD%5BAuthor%5D&cauthor=true&cauthor_uid=25485619), [Gentleman RC](http://www.ncbi.nlm.nih.gov/pubmed/?term=Gentleman%20RC%5BAuthor%5D&cauthor=true&cauthor_uid=25485619), [Manning G](http://www.ncbi.nlm.nih.gov/pubmed/?term=Manning%20G%5BAuthor%5D&cauthor=true&cauthor_uid=25485619), [Yauch RL](http://www.ncbi.nlm.nih.gov/pubmed/?term=Yauch%20RL%5BAuthor%5D&cauthor=true&cauthor_uid=25485619), [Bourgon R](http://www.ncbi.nlm.nih.gov/pubmed/?term=Bourgon%20R%5BAuthor%5D&cauthor=true&cauthor_uid=25485619), [Stokoe D](http://www.ncbi.nlm.nih.gov/pubmed/?term=Stokoe%20D%5BAuthor%5D&cauthor=true&cauthor_uid=25485619), [Modrusan Z](http://www.ncbi.nlm.nih.gov/pubmed/?term=Modrusan%20Z%5BAuthor%5D&cauthor=true&cauthor_uid=25485619), [Neve RM](http://www.ncbi.nlm.nih.gov/pubmed/?term=Neve%20RM%5BAuthor%5D&cauthor=true&cauthor_uid=25485619), [de Sauvage FJ](http://www.ncbi.nlm.nih.gov/pubmed/?term=de%20Sauvage%20FJ%5BAuthor%5D&cauthor=true&cauthor_uid=25485619), [Settleman J](http://www.ncbi.nlm.nih.gov/pubmed/?term=Settleman%20J%5BAuthor%5D&cauthor=true&cauthor_uid=25485619), [Seshagiri S](http://www.ncbi.nlm.nih.gov/pubmed/?term=Seshagiri%20S%5BAuthor%5D&cauthor=true&cauthor_uid=25485619), [Zhang Z](http://www.ncbi.nlm.nih.gov/pubmed/?term=Zhang%20Z%5BAuthor%5D&cauthor=true&cauthor_uid=25485619). A comprehensive transcriptional portrait of human cancer cell lines. [Nat Biotechnol.](http://www.ncbi.nlm.nih.gov/pubmed/25485619) 2015;33:306-12.
